# Technological diversification and structural persistence in Peru's patent and utility model filings, 2000–2024

**DOI:** 10.3389/frma.2026.1935094

**Published:** 2026-09-30

**Authors:** David Arratea-Pillco, José Gabriel Seijas-Díaz, Enrique Alejandro Barbachan-Ruales, Julio Iván Torres-Manrique, Italo W. Alejos-Patiño, Marco Arratea-Pillco

**Affiliations:** 1 Universidad Nacional de San Agustín de Arequipa (UNSA), Arequipa, Peru; 2 Universidad Nacional Autónoma de Alto Amazonas (UNAAA), Yurimaguas, Peru; 3 Universidad Nacional de Educación Enrique Guzmán y Valle (UNE), Lima, Peru; 4 Universidad Nacional Hermilio Valdízan (UNHEVAL), Huánuco, Peru

**Keywords:** innovation indicators, international patent classification, patent analytics, Peru, research metrics, structural persistence, technological diversification, utility models

## Abstract

**Introduction:**

Aggregate patent counts provide information on filing volume but do not reveal whether a national filing corpus is technologically concentrated, diversified, or structurally reordered over time. This study examines the International Patent Classification (IPC) composition of Peru's patent and utility model filing corpus and distinguishes distributional diversification from structural reordering.

**Methods:**

We analyzed 32,966 IPC-classified patent and utility model applications filed before INDECOPI between 2000 and 2024, distributed across 131 primary IPC classes. An integrated descriptive patent analytics framework combined concentration, diversity, dominance, rank persistence, set overlap, distributional distance, cumulative hierarchy, legal-instrument robustness, and applicant-origin contextual indicators.

**Results:**

The filing distribution became less concentrated and more even over time. Across predefined 5-year windows, mean annual HHI decreased from 0.220 in 2000–2004 to 0.072 in 2020–2024, while mean Shannon entropy increased from 3.670 to 5.022 bits. Mean Top-1, Top-3, and Top-10 shares also declined. Nevertheless, A61, C07, and A01 together accounted for 49.5% of the 2000–2024 panel. Spearman correlations across consecutive periods ranged from 0.887 to 0.941 in the fixed 131-class universe, while robustness checks using alternative class universes supported the same general interpretation, although with lower coefficients. Instrument-stratified analyses indicated that the diversification pattern was not solely attributable to combining patents and utility models.

**Discussion:**

The results indicate diversification without full structural reordering. The study characterizes an office-based jurisdictional filing corpus before INDECOPI and should not be interpreted as direct evidence of domestic technological capability, patent quality, or innovation performance. The framework provides a reproducible approach for distinguishing broader IPC-classified filing participation from deeper changes in technological hierarchy.

## 1 Introduction

Patent data are a central source for studying technological activity because patent documents contain structured information on applications, applicants, jurisdictions, filing dates, legal instruments, technological classifications, and technical subject matter. For this reason, patents have long been used in research metrics, innovation studies, technology management, and patent analytics to examine technological trajectories, sectoral specialization, knowledge diffusion, and the organization of inventive activity. However, patent indicators must be interpreted with caution. Patents do not measure innovation directly, and they do not automatically represent technological capability, commercialization, or economic value. Their meaning depends on legal systems, applicant strategies, filing practices, examination procedures, sectoral propensities to patent, and counting conventions. This caution has been emphasized in classic work on patent statistics and remains central in contemporary patent analytics, where the increasing availability of machine-readable patent data has expanded analytical possibilities but also increased the need for careful corpus definition and interpretation (Abbas et al., 2014; Aristodemou and Tietze, 2018; Chuprat et al., 2025; Griliches, 1990; Jiang and Goetz, 2025; Mejia and Kajikawa, 2025; Narin et al., 1987; Shomee et al., 2025).

A key limitation of aggregate patent counts is that they describe volume but not internal technological structure. Annual totals can show whether filing activity increased or decreased, but they do not reveal whether filings are concentrated in a small number of technological domains, broadly distributed across many fields, or persistently organized around the same dominant classes. Similarly, a decline in concentration does not necessarily imply deep structural change, and an increase in filing volume does not necessarily indicate broader technological diversification. Aggregate counts are also sensitive to the type of application counted, the jurisdiction where applications are filed, whether records are counted by office or by origin, and whether the analysis uses applications, grants, priority filings, families, or citations. These issues are well recognized in the patent statistics literature and in international guidance on patent indicators, which stress that patent data require explicit decisions about counting rules, coverage, comparability, and interpretive scope (de Rassenfosse et al., 2013; Griliches, 1990; OECD, 2009; Squicciarini et al., 2013). Therefore, studying a national filing corpus requires moving beyond aggregate totals toward indicators that capture concentration, diversity, dominance, persistence, and compositional change.

The International Patent Classification (IPC) provides a standardized basis for examining this internal technological structure. The IPC is a hierarchical classification system administered by the World Intellectual Property Organization and used internationally to classify patent documents according to technological subject matter. At the class level, IPC symbols are identified by a section letter and a two-digit class number, such as A61, C07, or B65. This level of aggregation is less detailed than subclasses, groups, or subgroups, but it offers a practical balance between interpretability and statistical stability for longitudinal analysis. IPC-based approaches have been widely used to construct technological profiles, compare country-level structures, map patent portfolios, and examine knowledge spaces. They are particularly useful when the research objective is not to evaluate individual patents, but to characterize the distribution of filings across technological domains. In this study, the IPC class level provides the analytical lens for assessing whether Peru's patent and utility model filing corpus became more diversified over time and whether such diversification was accompanied by reordering of the dominant technological hierarchy (Kogler et al., 2013; Leydesdorff et al., 2014; Schmoch, 2008; World Intellectual Property Organization, 2024).

Distributional diversification and structural reordering should be treated as related but distinct phenomena. A filing corpus may become more diversified if applications are distributed across a larger number of IPC classes or if the distribution becomes more even. However, the same corpus may still preserve a stable technological hierarchy if the leading classes remain dominant and retain similar rank positions over time. This distinction is important because different indicators answer different analytical questions. Concentration and entropy summarize how uneven or dispersed the distribution is; Top-k shares show the extent of dominance by leading classes; rank correlations assess whether the ordering of classes persists; set-overlap measures show whether the same classes remain among the dominant groups; and distributional-distance measures capture broader compositional change. In this sense, distributional diversification does not necessarily imply structural reordering. This distinction builds on broader work showing that diversity is multidimensional, that technological variety may be related or unrelated, and that diversification can coexist with persistent knowledge structures and technological competencies (Balland et al., 2019; Frenken et al., 2007; Leten et al., 2007; Shannon, 1948; Stirling, 2007).

A further interpretive issue concerns applicant origin. Applications filed before a national intellectual property office include both resident and non-resident applicants. This distinction is especially important in economies where a large share of patent applications may be filed by foreign applicants seeking protection in the domestic jurisdiction. Therefore, a national office filing corpus represents a jurisdictional structure of filings, not automatically a resident technological profile. Applicant-origin composition conditions interpretation because the same IPC distribution may reflect domestic applicant activity, foreign filing strategies, or a combination of both. International patent statistics distinguish between office-based and origin-based perspectives, and WIPO defines origin according to the residence of the first named applicant. WIPO's World Intellectual Property Indicators and IP Statistics Data Center provide the main international framework for comparing resident and non-resident patent applications, while World Bank indicators provide additional country-level series. For this reason, the present study treats applicant-origin context as necessary for interpretation but does not equate the INDECOPI filing corpus with domestic technological capacity (de Rassenfosse et al., 2013; OECD, 2009; World Intellectual Property Organization, 2024; World Bank, 2024).

Peru provides a relevant case for examining these issues because its administrative patent and utility model records offer a basis for longitudinal IPC analysis, but systematic evidence on the internal technological structure of its filing corpus remains limited. Existing aggregate statistics can describe the number of applications filed before the national office, but they do not show whether the corpus became more or less concentrated, whether dominant IPC classes persisted, or whether diversification implied a reordering of the technological hierarchy (Abbas et al., 2014; Aristodemou and Tietze, 2018). The period 2000–2024 allows long-run observation while avoiding the partial-year bias associated with 2025. The joint analysis of patents and utility models is appropriate for characterizing the IPC-classified filing corpus before INDECOPI because both legal instruments receive IPC primary class codes, while the interpretation remains limited to jurisdictional filing structure rather than resident technological capability. This framing is consistent with patent analytics approaches that emphasize careful corpus construction, classification, validation, and interpretation before drawing broader conclusions from patent data (Chuprat et al., 2025; Mejia and Kajikawa, 2025).

This study asks whether the IPC composition of Peru's patent and utility model filing corpus became more diversified between 2000 and 2024, and whether such diversification was accompanied by structural reordering of the dominant IPC hierarchy. The analysis focuses on applications filed before INDECOPI and does not treat these records as direct measures of domestic technological capacity. To address this question, the study combines concentration, diversity, dominance, persistence, set-overlap, distributional-distance, cumulative-hierarchy, and applicant-origin-context indicators within a single descriptive patent analytics framework.

The article makes three contributions. Empirically, it provides a longitudinal IPC-based analysis of Peru's patent and utility model filing corpus for 2000–2024. Methodologically, it combines HHI, normalized HHI, Shannon entropy, Top-k dominance, Spearman rank correlation, Kendall's tau-b, Jaccard similarity, Jensen–Shannon distance, and cumulative concentration zones to distinguish broader IPC participation from deeper hierarchy displacement. Interpretively, it separates distributional diversification, structural persistence, and applicant-origin context, thereby reducing the risk of conflating jurisdictional patent activity with domestic technological capability. By distinguishing distributional diversification from structural reordering, the study provides a reproducible framework for interpreting national filing corpora without conflating jurisdictional patent activity with domestic technological capability.

## 2 Materials and methods

### 2.1 Study design and analytical objective

This study used an observational, retrospective, longitudinal, descriptive-analytical design to examine the technological structure of Peru's patent and utility model filing corpus. The original unit of observation was the individual application record contained in the INDECOPI administrative database, whereas the analytical units were the annual and period-level distributions of filings across primary International Patent Classification (IPC) classes. Period-level analyses used equal 5-year windows to reduce annual volatility while preserving temporal comparability across the 2000–2024 panel. The filing year recorded in the administrative database was used as the temporal reference, rather than publication, examination, or grant year.

The analysis was designed to characterize concentration, diversity, dominance, rank persistence, Top-k overlap, and compositional change in IPC-class distributions over time. Patent and utility model records are treated as informative but imperfect indicators of technological activity, requiring careful attention to counting conventions, institutional coverage, legal-instrument scope, and applicant populations (de Rassenfosse et al., 2013; Griliches, 1990; OECD, 2009). Consistent with patent analytics approaches that emphasize structured data preparation, classification, and distributional analysis (Abbas et al., 2014; Aristodemou and Tietze, 2018), no causal identification strategy was applied. All indicators are interpreted as descriptive measures of the structure of the office-based filing corpus.

### 2.2 Data source, jurisdiction, and analytical corpus

The primary data source was the historical administrative database of Peru's national industrial property authority, INDECOPI, provided under Memorandum No. 000102-2026-DIN/INDECOPI. The administrative extract covered intellectual property records filed in Peru through 2025 and originally contained three administrative subsystems: patents and utility models, industrial designs, and plant variety certificates. The analytical corpus for this study was restricted to patent and utility model applications because these legal instruments receive IPC codes suitable for technological-structure analysis.

Industrial designs were excluded because they are classified under the Locarno Classification, whereas plant variety certificates were excluded because they are governed by plant-variety protection categories rather than IPC technological classes. The IPC provides an internationally standardized hierarchical classification system for patent documents and is widely used for technology mapping, portfolio analysis, and country-level comparisons (Leydesdorff et al., 2014; Schmoch, 2008; World Intellectual Property Organization, 2024).

The main analytical panel was limited to applications filed between 2000 and 2024. Records from 2025 were excluded because that year was incomplete at the time of extraction and was therefore not part of the longitudinal analytical window. Aggregate World Bank data were used only for regional contextualization of resident and non-resident patent applications. These external data provide country-level applicant-origin counts but do not reproduce the INDECOPI IPC-class panel. The regional data were used only for contextualization and were not merged with the INDECOPI IPC-level analytical panel.

### 2.3 Record flow, data quality, and audit procedures

The validated administrative database contained 43,107 records. Industrial design records, corresponding to 7,675 records, were excluded because they are not classified using the International Patent Classification. Plant variety certificate records, corresponding to 932 records, were excluded because they follow plant-variety protection categories rather than IPC technological classes. After these exclusions, the eligible patent and utility model corpus comprised 34,500 records, consisting of 29,165 patent applications and 5,335 utility model applications. Patent and utility model counts are mutually exclusive components of the eligible corpus and are not additional exclusions.

The main analytical panel was then restricted to applications filed between 2000 and 2024, yielding 32,966 records. The remaining 1,534 records from 2025 were excluded from the main panel because 2025 was incomplete at the time of extraction. Data quality checks were conducted before indicator construction. Filing years were verified for completeness, primary IPC classes were validated against the expected IPC class format, and duplicate application-year-IPC combinations were checked using the application identifier, filing year, and primary IPC class. No records in the 2000–2024 analytical panel were excluded because of missing filing year, invalid primary IPC class, or duplicate application-year-IPC combinations. The final panel contained 131 distinct primary IPC classes. The record-flow audit is reported in Table 1.

**Table 1:** Record flow from the INDECOPI administrative database to the analytical panel.

Stage	Records	Note
Validated administrative database as received	43,107	Historical administrative extract provided by INDECOPI
Excluded industrial designs	−7,675	Excluded because industrial designs are not IPC-classified
Excluded plant variety certificates	−932	Excluded because plant variety certificates are not IPC-classified
Eligible patent and utility model corpus, 2000–2025	34,500	Patent and utility model applications with valid filing year and primary IPC class
Patent applications within eligible corpus	29,165	Mutually exclusive component of the eligible corpus
Utility model applications within eligible corpus	5,335	Mutually exclusive component of the eligible corpus
Main analytical panel, 2000–2024	32,966	Used for all main manuscript tables and figures
Excluded partial year 2025	1,534	Excluded because 2025 was incomplete at the time of extraction
Distinct primary IPC classes, 2000–2024	131	Fixed universe used for persistence and distributional-distance analyses
Records excluded for missing filing year	0	Filing year required for longitudinal analysis
Records excluded for invalid or missing primary IPC class	0	IPC class must match section A–H plus two digits
Duplicate application-year-IPC combinations	0	Checked using application identifier, filing year, and primary IPC class

The validated database contained 43,107 records. After excluding 7,675 industrial designs and 932 plant variety certificates, the eligible patent and utility model corpus comprised 34,500 records. Of these, 32,966 records filed during 2000–2024 constituted the main analytical panel, whereas 1,534 records from the partial year 2025 were excluded because 2025 was incomplete at the time of extraction. Patent and utility model counts are mutually exclusive components of the eligible corpus and are not additional exclusions. All longitudinal results reported for 2000–2024 use n = 32,966 as the denominator.

### 2.4 IPC harmonization and classification level

IPC codes were harmonized before analysis to ensure consistency across records and years. Classification strings were standardized to uppercase, non-informative spacing was removed, and delimiters were normalized only for parsing purposes. The original order of IPC symbols within each classification string was preserved throughout the extraction process; no alphabetical or numerical sorting of IPC symbols was applied before identifying the analytical class. IPC class was defined as the section letter plus two-digit class number, such as A61, C07, or B65. This level is distinct from the IPC subclass level, which adds a further letter, and from more detailed main-group or subgroup levels.

The administrative extract did not contain explicit WIPO Standard ST.8 invention-information or additional-information flags, nor an independent field identifying a formally designated primary IPC symbol. Therefore, the primary IPC class was operationalized as the class of the first IPC symbol recorded in the original classification string. This rule was applied deterministically to all records before reducing IPC symbols to the class level. The extraction rule therefore used the first recorded symbol as provided in the source field, not the alphabetically smallest IPC class after parsing.

The analysis was conducted at the IPC class level because it provides an interpretable balance between technological specificity and sufficient cell size for longitudinal comparison. The final 2000–2024 panel contained 131 observed primary IPC classes across all eight IPC sections. Using the class level reduced sparsity relative to subclass- or group-level analysis and enabled the construction of stable annual and period-level share distributions. This choice is consistent with prior IPC-based approaches that use patent classifications to describe technological portfolios, map technological profiles, and compare country-level or regional structures (Leydesdorff et al., 2014; Schmoch, 2008). Because patent classification systems and administrative practices may evolve over time, the observed IPC class universe was treated as an empirical representation of the INDECOPI filing corpus rather than as a complete measure of all possible technological relationships (Lafond and Kim, 2019; World Intellectual Property Organization, 2024).

### 2.5 Unit of analysis and primary IPC class approach

Each application record was assigned to a single primary IPC class for the main analysis. This one-record-one-primary-class approach preserves mutually exclusive annual and period-level IPC distributions, avoids double counting of applications with multiple IPC assignments, and permits the calculation of concentration, diversity, dominance, rank persistence, set-overlap, and distributional-distance indicators on comparable share vectors. The approach is therefore appropriate for describing the primary IPC-classified filing distribution of the corpus. However, it is a deliberate simplification: patent and utility model applications may contain additional IPC symbols that reflect cross-domain or multidisciplinary technological content. Consequently, the results should be interpreted as primary-class filing distributions rather than as all-IPC profiles, co-classification networks, or full technological relatedness structures.

Because the primary IPC construction is central to the analysis, diagnostic checks were conducted to assess whether the first-symbol rule could have introduced an alphabetical sorting artifact. In the 2000–2024 analytical panel, the selected primary IPC class matched the class of the first listed IPC symbol in all 32,966 records. The panel included 17,656 records with more than one IPC symbol, representing 53.56% of the corpus, and 14,417 records with more than one IPC class, representing 43.73% of the corpus. Among records with more than one IPC class, 6,005 records, or 41.65%, had a selected primary IPC class that was not the alphabetically smallest class on the record. This diagnostic provides evidence against a strict alphabetical sorting artifact in the construction of the primary IPC class. The same diagnostic was also calculated separately for patent applications and utility model applications and is reported in the Supplementary material.

The primary-class approach was retained because it provides a transparent and reproducible counting convention for the main descriptive analysis. At the same time, the diagnostic results indicate that the corpus contains a substantial multi-IPC component, especially among patent applications, and that the primary-class distribution should not be interpreted as capturing the full technological content of each filing. Future extensions could examine all IPC assignments, fractional counting, IPC co-classification networks, or subclass-level structures if complete and consistently structured secondary-classification data become available, as such approaches would address different aspects of technological relatedness and portfolio structure (Balland et al., 2019; Leydesdorff et al., 2014; Schmoch, 2008).

### 2.6 Concentration, diversity, and dominance metrics

For each year and analytical period, the IPC-class distribution was represented as a vector of class shares. Let xi denote the number of filings assigned to IPC class i, let X denote the total number of filings in the corresponding year or period, and let pi = xi/X denote the share of filings assigned to IPC class i. Let n denote the number of active IPC classes in the corresponding year or period.

Concentration was measured using the Herfindahl-Hirschman Index and its normalized version. The Herfindahl-Hirschman Index was calculated as the sum of squared IPC-class shares. The normalized HHI was calculated to account for differences in the number of active IPC classes across years or periods. In this study, HHI was used only as a concentration measure for IPC-class shares and was not interpreted as market concentration, technological maturity, or innovation performance.

Diversity was measured using Shannon entropy in bits. Shannon entropy captures the evenness or dispersion of the IPC-class distribution and was calculated from IPC-class shares using logarithms in base 2 (Shannon, 1948; Stirling, 2007). Classes with zero share in a given year or period contributed zero to the entropy sum by convention. Entropy was not interpreted as technological quality, inventive capability, or domestic innovation capacity.

Dominance was assessed using cumulative Top-k shares, specifically Top-1, Top-3, Top-5, and Top-10. These indicators report the percentage of filings accounted for by the most frequent IPC classes in each year or period. Together, HHI, normalized HHI, Shannon entropy, and Top-k shares describe complementary aspects of the same IPC-share distribution: concentration, evenness, and dominance by leading classes.

### 2.7 Persistence and structural change metrics

Persistence and structural change were evaluated across five predefined analytical periods: 2000–2004, 2005–2009, 2010–2014, 2015–2019, and 2020–2024. Five-year periods were used to balance temporal resolution and statistical stability. This aggregation reduces the influence of short-term annual fluctuations, keeps all analytical periods equal in duration, and enables direct comparison of period-level IPC-share distributions. The final period ends in 2024 because 2025 was incomplete at the time of extraction and was therefore excluded from the longitudinal analytical panel.

For the main persistence analysis, period-level IPC-share vectors were constructed over the fixed universe of 131 primary IPC classes observed in the 2000–2024 analytical panel. Classes with no filings in a given period were assigned a share of zero, allowing entry, exit, and low-frequency classes to remain part of the comparison rather than restricting the analysis only to classes active in both periods. This fixed-universe specification preserves the full observed filing repertoire and avoids dropping classes that appear, disappear, or remain peripheral over time. However, it can also increase apparent rank stability when many classes are persistently inactive or have very low shares. For this reason, fixed-universe rank correlations were interpreted jointly with Top-k overlap, Jensen-Shannon distance, and robustness checks based on alternative class universes.

To assess the sensitivity of the persistence results to the analytical universe, robustness analyses were conducted using six additional specifications. First, a union-active universe included IPC classes active in either of the two periods being compared. Second, an intersection-active universe included only IPC classes active in both periods. Third, Top-20 and Top-50 universes were constructed from the union of the highest-ranked classes in each pair of consecutive periods. Fourth, two filing-threshold universes retained classes with at least 10 or at least 25 filings across the two periods being compared. These alternative specifications were used to determine whether the observed rank persistence was driven primarily by inactive or low-share classes in the fixed 131-class universe. The full robustness results are reported in Supplementary Table S3.

Spearman rank correlation was used as the main indicator of rank-order persistence, and Kendall's tau-b was included as a complementary rank-association measure because it is suitable when tied ranks are present. Continuity in the membership of dominant IPC-class sets was assessed using Jaccard similarity for Top-5, Top-10, and Top-20 sets. IPC classes were ranked by filing share in descending order; when counts were tied, IPC class codes were ordered alphanumerically to ensure deterministic reproducibility. Boundary diagnostics were conducted to identify whether ties crossed Top-k cut-offs, and these diagnostics are reported in the Supplementary material.

Compositional change was assessed using Jensen-Shannon distance, derived from Jensen-Shannon divergence, a symmetric information-theoretic measure for comparing probability distributions (Endres and Schindelin, 2003; Lin, 1991). Jensen-Shannon distance was computed on period-level IPC-share distributions using logarithms in base 2. The implementation used scipy.spatial.distance.jensenshannon with base = 2, which returns the Jensen-Shannon distance, not the divergence. Jensen-Shannon distance was interpreted comparatively across adjacent and non-adjacent period pairs rather than through uncalibrated qualitative thresholds such as low, moderate, or high.

Jaccard similarity was calculated as the size of the intersection between two Top-k IPC-class sets divided by the size of their union. Spearman's rho and Kendall's tau-b were calculated using period-level IPC-share vectors. Jensen-Shannon distance was calculated from the Jensen-Shannon divergence between two period-level IPC-share distributions and their midpoint distribution. These indicators were interpreted descriptively: rank correlations measure persistence in class ordering, Jaccard similarity measures overlap in dominant class membership, and Jensen-Shannon distance measures overall compositional difference between IPC-share distributions.

Statistical *p*-values associated with rank correlations were reported as threshold values, for example *p* < 0.001, rather than as zero. Because the analysis covers the validated administrative panel rather than a probability sample, *p*-values were treated as complementary information only. Interpretation focused primarily on coefficient magnitude, direction, stability across specifications, Top-k overlap, and Jensen-Shannon distance.

### 2.8 Cumulative IPC concentration zones

To characterize the cumulative hierarchy of the IPC-class distribution, all IPC classes in the 2000–2024 analytical panel were ranked in descending order according to their total number of records. The cumulative filing share was then calculated across this ordered distribution. Four descriptive concentration zones were defined using cumulative filing-share thresholds: Core, corresponding to the first 0–50% of cumulative filings; Intermediate, corresponding to 50–80%; Peripheral, corresponding to 80–95%; and Long-tail, corresponding to 95–100%.

These thresholds were used as a transparent presentational device to summarize the highly uneven cumulative distribution into high-share, mid-share, peripheral, and long-tail portions of the corpus. They should not be interpreted as validated typological boundaries, statistical clusters, or network-derived communities. Because IPC classes are discrete units, cumulative zone shares do not necessarily equal the threshold values exactly. A conservative do-not-cross rule was applied consistently at each boundary: an IPC class was assigned to a zone only if its inclusion did not move the cumulative share beyond the upper threshold of that zone; the next IPC class started the following zone. This explains why the Core zone reached 49.5% rather than exactly 50.0%.

The labels Core, Intermediate, Peripheral, and Long-tail are therefore descriptive cumulative concentration categories and do not denote a network-based core-periphery model. No co-classification network, centrality measure, community-detection procedure, or modularity analysis was estimated. The purpose of this classification was to summarize how much of the filing corpus was accounted for by high-share, mid-share, and low-share IPC classes using a reproducible cumulative-share rule.

### 2.9 Scope of the combined legal-instrument corpus

Patent applications and utility model applications were analyzed jointly in the main analysis because both legal instruments receive IPC codes and the objective of the study was to characterize the IPC-classified filing corpus before INDECOPI. This combined-corpus approach provides a unified view of the jurisdictional filing structure while preserving a consistent analytical unit across years, periods, and IPC classes. However, patents and utility models are distinct legal instruments, and their filing patterns may differ by applicant type, technological field, examination procedure, inventive-step requirements, and strategic use.

To address this issue, instrument-stratified robustness analyses were conducted in addition to the combined-corpus analysis. Annual filing counts and period-level concentration, diversity, dominance, and persistence indicators were calculated separately for patent applications, utility model applications, and the combined corpus. The 2000–2024 analytical panel contained 32,966 records, of which 28,048 were patent applications and 4,918 were utility model applications. Instrument-stratified indicators were used to assess whether the diversification pattern observed in the combined corpus was also present within patent-only and utility-model-only subsets, or whether it was mainly driven by changes in the relative composition of legal instruments.

The main text reports the combined-corpus results because the article's primary objective is to characterize the IPC-classified filing structure before INDECOPI. The instrument-stratified results are used as robustness evidence and are reported in the Supplementary material. Accordingly, the results should be interpreted as describing the jurisdictional IPC-classified filing corpus, with additional checks showing whether the main patterns are consistent across legal instruments. They should not be interpreted as evidence that patents and utility models have identical legal, technological, or strategic meanings.

### 2.10 Regional contextualization

To contextualize the applicant-origin composition of Peru's patent filing environment, aggregate patent-application data were compiled for Brazil, Chile, Colombia, Mexico, and Peru using World Bank World Development Indicators (World Bank, 2024). The indicators used were patent applications by residents and patent applications by non-residents. These data were used only for regional applicant-origin context and were not merged with the INDECOPI IPC-level administrative panel. The regional comparison does not include utility models, does not provide IPC-class distributions, and does not identify whether specific IPC classes in the INDECOPI corpus were driven by resident or non-resident applicants.

The regional table was reconstructed using two clearly defined comparison windows. The first window reports annual means for 2000–2005. The second reports the latest available WDI year in the supplied resident and non-resident patent-application series, which was 2021 for the countries included in the comparison. The most recent comparison is therefore reported as “2021 latest available WDI year” rather than as a 2021–2024 period. Resident share was calculated as resident patent applications divided by total patent applications, where total patent applications equals resident plus non-resident applications. The non-resident/resident ratio was calculated as non-resident patent applications divided by resident patent applications. The term non-resident/resident ratio is used descriptively and replaces the more interpretively loaded term dependency ratio.

These regional indicators were treated strictly as contextual evidence. They are independent from the 5-year IPC analytical periods used for the INDECOPI panel and were not used to validate, adjust, or replace the IPC-based results. Because patent counts are sensitive to jurisdiction, institutional coverage, applicant origin, legal instrument, and counting convention, the regional comparison is used only to support interpretation of the jurisdictional filing corpus in relation to applicant-origin composition (de Rassenfosse et al., 2013; Griliches, 1990; World Intellectual Property Organization, 2024; World Bank, 2024).

### 2.11 Computational reproducibility and open science

All data processing, indicator construction, robustness analyses, table generation, figure production, and validation checks were implemented in Python. The final workflow was executed in Google Colab on a Linux x86_64 environment using Python 3.12.13. The main libraries used for the analysis included pandas 2.2.2 and NumPy 2.0.2 for data processing, SciPy 1.16.3 for rank-correlation and Jensen-Shannon distance calculations, Matplotlib 3.10.0 for figure generation, and openpyxl 3.1.5 for Excel output and manuscript-ready tables.

The computational workflow generated the record-flow audit, the year-by-IPC analytical panel, annual indicators, period-level indicators, instrument-stratified robustness checks, persistence robustness analyses across alternative class universes, primary-IPC diagnostic checks, cumulative concentration zones, reconstructed regional applicant-origin tables, manuscript tables, figures, and validation outputs. Reproducibility checks included verification that annual record counts summed to the final 2000–2024 analytical panel of 32,966 records, confirmation that 2025 records were excluded from the main longitudinal analysis, validation that the fixed IPC-class universe contained 131 primary IPC classes, checks that IPC share vectors summed to one within each year or period, confirmation that Jensen-Shannon distances were computed with logarithms in base 2, replacement of zero-printed rank-correlation *p*-values with threshold notation, and audit checks for the primary IPC extraction rule.

To support reproducibility without redistributing restricted administrative microdata, a derived reproducibility package was prepared for this study (Arratea Pillco et al., 2026). The package includes aggregated year-by-IPC count matrices, annual and period-level indicator tables, instrument-stratified robustness tables, persistence robustness outputs, primary-IPC diagnostic summaries, regional annual source series, reconstructed regional comparison tables, codebook files, a README file, software-environment information, validation outputs, and an executable script that regenerates the core aggregate workflow from the public aggregated inputs, including annual indicators, 5-year-period averages, the Jensen-Shannon distance matrix, and the cumulative IPC distribution. Additional robustness tables, tie diagnostics, primary-IPC diagnostics, regional audit outputs, manuscript tables, and figures are provided as derived validation outputs in the package. Some diagnostics were generated from restricted administrative fields that are not redistributed publicly, including original IPC classification strings and application-level identifiers. The package does not include applicant names, application identifiers, full administrative records, or unrestricted copies of the raw INDECOPI database.

The raw INDECOPI administrative database is subject to institutional access conditions and is not publicly redistributed by the authors. Derived aggregate materials, robustness outputs, codebook files, software-environment information, validation outputs, and analysis scripts supporting this study have been deposited in Zenodo. The all-versions DOI is https://doi.org/10.5281/zenodo.22070845, and the corrected version v1.1 is available at https://doi.org/10.5281/zenodo.22178836. The deposited reproducibility package does not include applicant names, application identifiers, full administrative records, or unrestricted copies of the raw INDECOPI database. Supplementary Tables S1–S5 and Supplementary File S1 are also provided with the manuscript submission. Requests for access to the underlying source database should be directed to INDECOPI through its applicable administrative procedures. This approach follows FAIR-oriented principles for improving the findability, accessibility, interoperability, and reuse of derived research outputs while recognizing legitimate restrictions on administrative source data (Wilkinson et al., 2016).

## 3 Results

### 3.1 Corpus evolution and IPC breadth

Table 2 and Figure 1 summarize the annual evolution of the 2000–2024 analytical panel. The corpus contains 32,966 IPC-classified patent and utility model records distributed across 131 primary IPC classes. Annual filings ranged from 370 records in 2010 to 1,949 records in 2024, while the number of active IPC classes ranged from 65 in 2002 to 104 in both 2023 and 2024.

**Table 2:** Annual IPC concentration, diversity, and dominance indicators for Peru's patent and utility model corpus (2000–2024).

Year	*N*	Active classes	HHI	HHI norm	Shannon entropy bits	Top 1%	Top 3%	Top 5%	Top 10%
2000	1122	86	0.158	0.148	4.05	27.9	58.1	63.5	73.2
2001	1014	75	0.201	0.19	3.633	33.2	65.6	70.4	78.9
2002	895	65	0.266	0.254	3.397	49.4	65.4	70.7	80.2
2003	962	73	0.263	0.253	3.501	49.3	64.3	68.7	77.7
2004	902	76	0.21	0.199	3.767	42.9	60.3	66.4	76.6
2005	1098	74	0.248	0.238	3.426	46	67.9	72.7	80.4
2006	1301	83	0.267	0.258	3.406	49	69.3	72.8	79.7
2007	1385	79	0.253	0.244	3.481	47.5	67.2	71.6	79.8
2008	1559	83	0.248	0.239	3.519	46.6	67.1	71.1	78.1
2009	762	79	0.194	0.184	3.944	40.7	61.2	64.7	72.2
2010	370	67	0.101	0.088	4.709	28.9	41.4	47.6	61.1
2011	1240	88	0.163	0.154	4.176	37.4	54.5	60.6	71.9
2012	1310	100	0.134	0.125	4.501	34.1	47.6	54	66.4
2013	1385	94	0.095	0.085	4.78	25.8	44.5	52.2	62.8
2014	1461	96	0.079	0.069	4.937	20.4	41.3	49.5	60.7
2015	1436	99	0.088	0.079	4.819	19.7	43.9	51.7	63.7
2016	1391	98	0.08	0.07	4.942	19.8	42.5	48.1	59.5
2017	1472	101	0.079	0.069	4.991	21.5	41	46.9	58.8
2018	1463	98	0.081	0.072	4.919	21.9	41.6	48.5	60.8
2019	1545	99	0.068	0.059	5.075	19.4	38.8	45.3	57.9
2020	1660	98	0.074	0.065	4.985	17.8	40.8	47.5	59.2
2021	1459	96	0.084	0.074	4.834	19.7	42.4	49.3	62.1
2022	1879	98	0.082	0.072	4.876	20.1	43.2	50.3	63.3
2023	1946	104	0.066	0.057	5.113	18.3	38.3	45.1	59.5
2024	1949	104	0.053	0.044	5.3	15.1	34.3	41.5	54.8

n sums to 32,966 across the 25 years shown, matching the validated 2000–2024 main panel. HHI, Herfindahl-Hirschman Index; HHI_norm, normalized HHI; H, Shannon entropy in bits, calculated using base 2; Top-k, cumulative share of the k most active classes.

**Figure 1:**
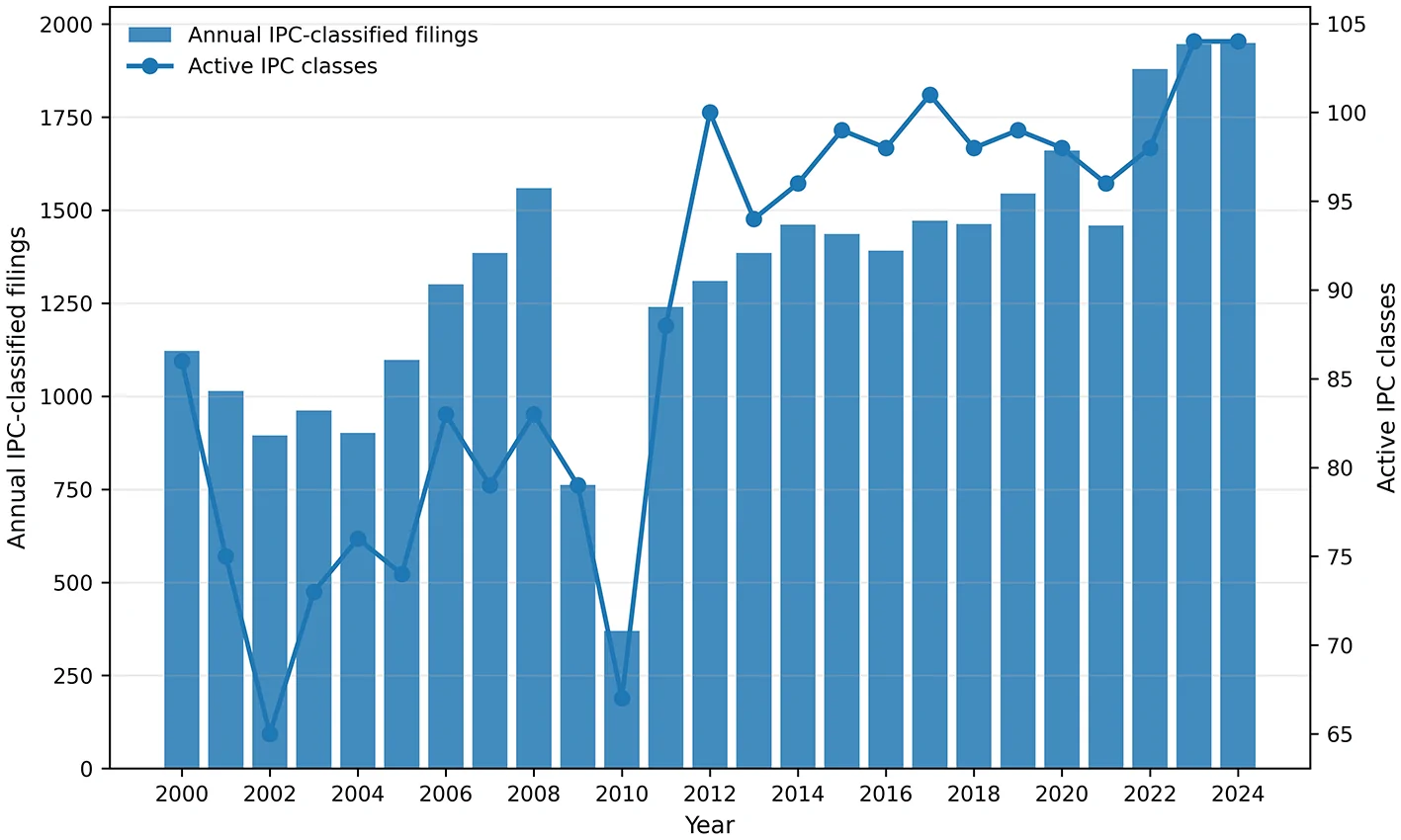
Annual IPC-classified patent and utility model filings and active IPC classes in Peru, 2000–2024.

The series shows a low-volume interval around 2009–2010, followed by recovery and expansion in both filings and active IPC classes. From 2011 onward, annual filing volumes remained above 1,200 records in most years and reached their highest levels in the final part of the series. IPC breadth also expanded during this later period. At this stage, the observed decline in 2009–2010 is treated descriptively; no administrative or institutional cause is inferred from the filing series alone.

### 3.2 Concentration and diversification

Table 2 and Figure 2 bring together the annual concentration, diversity, and dominance indicators for the combined patent and utility model filing corpus. Looking at the annual series, IPC-class concentration declines broadly while distributional evenness rises across the 2000–2024 analytical panel. Annual HHI peaked in 2006 and hit its lowest point in 2024, and Shannon entropy moved in the opposite direction, as expected. Rather than lean on a single peak-to-endpoint comparison, though, the interpretation that follows is anchored to predefined 5-year analytical windows.

**Figure 2:**
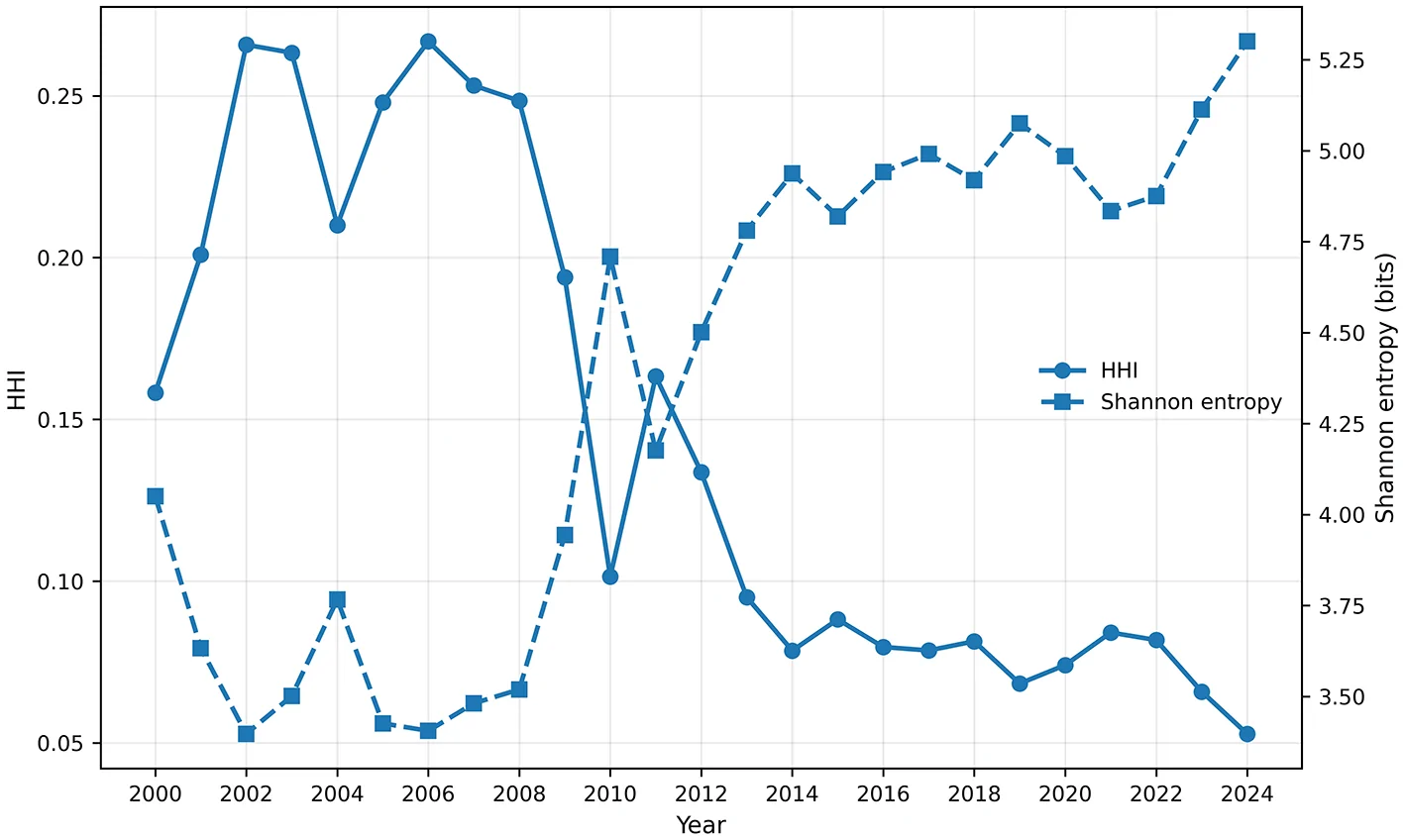
Annual HHI and Shannon entropy of Peru's IPC-classified patent and utility model filing corpus, 2000–2024.

Table 3 presents the annual indicator averages for each 5-year period. Mean annual HHI rose slightly, from 0.220 in 2000–2004 to 0.242 in 2005–2009, mirroring the highly concentrated structure of the early corpus, and then fell steadily — to 0.114 in 2010–2014, 0.079 in 2015–2019, and 0.072 in 2020–2024. Normalized HHI traced the same trajectory, dropping from 0.209 in 2000–2004 and 0.232 in 2005–2009 down to 0.062 in 2020–2024.

**Table 3:** Period-average annual IPC concentration, diversity, and dominance indicators, 2000–2024.

Period	Total *n*	Mean active classes	Mean HHI	Mean HHI norm	Mean Shannon entropy bits	Mean top-1%	Mean top-3%	Mean top-10%
2000–2004	4,895	75	0.22	0.209	3.67	40.54	62.74	77.31
2005–2009	6,105	79.6	0.242	0.232	3.555	45.94	66.55	78.03
2010–2014	5,766	89	0.114	0.104	4.621	29.33	45.86	64.58
2015–2019	7,307	99	0.079	0.07	4.949	20.48	41.54	60.17
2020–2024	8,893	100	0.072	0.062	5.022	18.21	39.79	59.79

Periods correspond to predefined 5-year analytical windows. Total n is the number of records filed within each period. Mean active classes, HHI, normalized HHI, Shannon entropy, and Top-k shares are annual averages calculated across the years within each period.

Shannon entropy tells a similar story, climbing clearly once the early high-concentration years give way. Mean annual entropy stood at 3.670 bits in 2000–2004 and 3.555 bits in 2005–2009, then rose to 4.621 bits in 2010–2014, 4.949 bits in 2015–2019, and 5.022 bits in 2020–2024. The average number of active IPC classes grew as well, from 75.0 in 2000–2004 to 100.0 in 2020–2024.

The dominance indicators point the same way. Mean Top-1 share fell from 40.54% in 2000–2004 and 45.94% in 2005–2009 to 18.21% in 2020–2024. Mean Top-3 share dropped from 62.74% in 2000-2004 and 66.55% in 2005–2009 to 39.79% in 2020–2024. Mean Top-10 share also declined, from 77.31% in 2000–2004 and 78.03% in 2005–2009 to 59.79% in 2020–2024. Together these shifts point to weaker dominance by the leading IPC classes, though the Top-10 group still made up more than half of annual filings, on average, in the most recent period.

Put together, the period-average results show that Peru's IPC-classified patent and utility model filings grew less concentrated, broader, and more evenly spread over time. That diversification, however, should be read as a shift in how filings are distributed across primary IPC classes — not as direct evidence of stronger technological capability, higher patent quality, or better domestic innovation performance.

### 3.3 Dominant IPC classes

Table 4 and Figure 3 show that the cumulative IPC-class distribution remained highly uneven over the 2000–2024 analytical panel. A61, corresponding to medical or veterinary science and hygiene, was the largest IPC class, with 9,264 records, representing 28.1% of the panel. C07, corresponding to organic chemistry, followed with 5,438 records, or 16.5%. Together, A61 and C07 accounted for 44.6% of all IPC-classified patent and utility model filings. When A01, corresponding to agriculture, forestry, and animal husbandry, was included, the cumulative share reached 49.5%, indicating that nearly half of the filing corpus was concentrated in only three IPC classes.

**Table 4:** Top 15 IPC classes in Peru's patent and utility model filing corpus, 2000–2024.

IPC_class_and_domain	Records	Share of panel (%)	Cumulative share (%)	Rank
A61 — Medical or veterinary science; hygiene	9264	28.1	28.1	1
C07 — Organic chemistry	5438	16.5	44.6	2
A01 — Agriculture; forestry; animal husbandry	1622	4.9	49.5	3
B65 — Conveying; packing; storing	895	2.7	52.2	4
A23 — Foods or foodstuffs	819	2.5	54.7	5
B01 — Physical or chemical processes	807	2.4	57.2	6
C12 — Biochemistry; microbiology	791	2.4	59.6	7
C22 — Metallurgy	666	2	61.6	8
A47 — Furniture; domestic articles	649	2	63.6	9
G01 — Measuring; testing	646	2	65.5	10
E21 — Earth or rock drilling; mining	507	1.5	67.1	11
E04 — Building	487	1.5	68.5	12
G06 — Computing; calculating	458	1.4	69.9	13
E02 — Hydraulic engineering; foundations	441	1.3	71.3	14
H04 — Electric communication technique	429	1.3	72.6	15

IPC domain descriptions follow the WIPO Guide to the International Patent Classification. Shares are calculated over the 2000–2024 analytical panel of 32,966 patent and utility model records.

**Figure 3:**
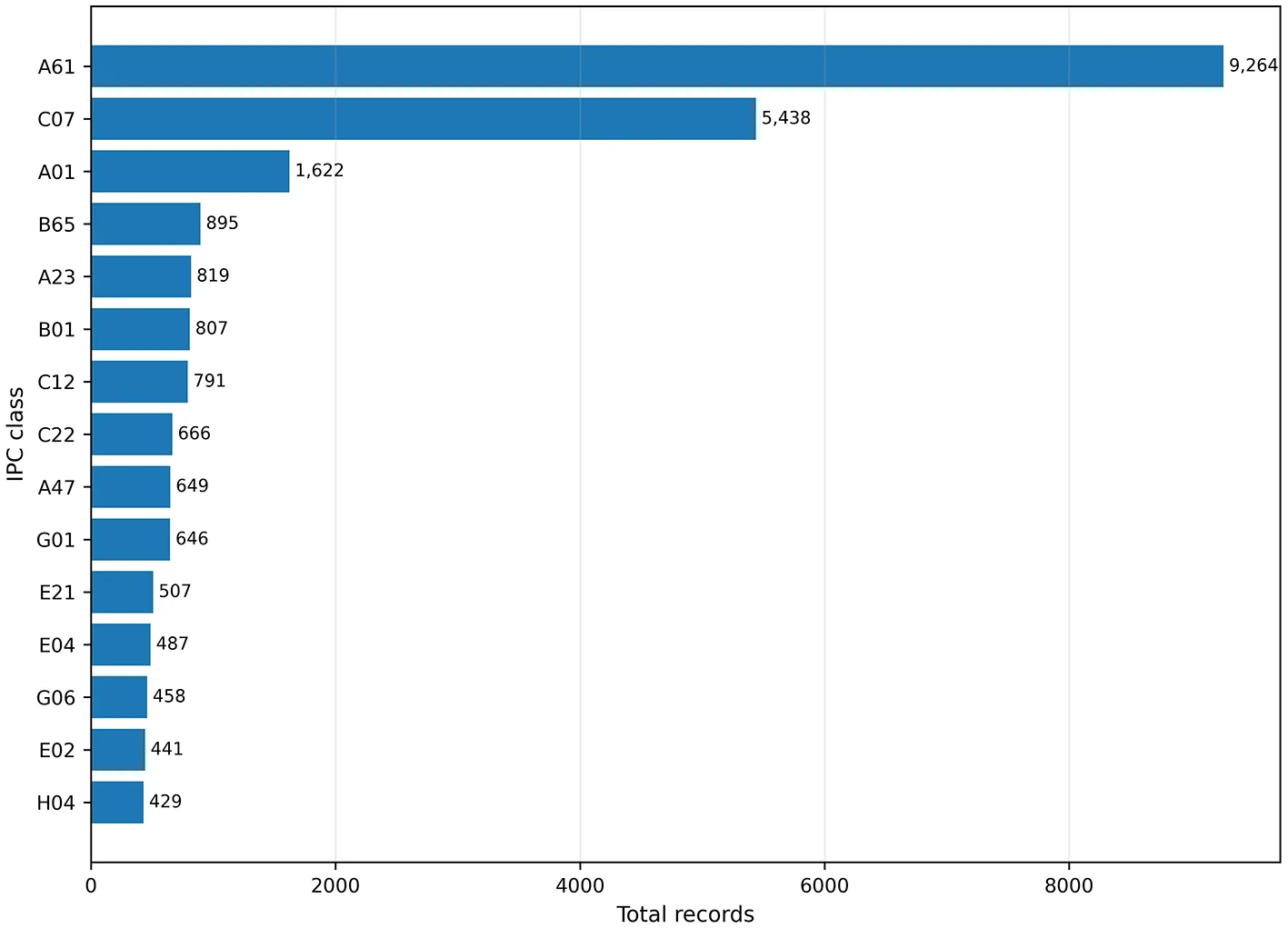
Top 15 IPC classes by total records in Peru's patent and utility model filing corpus, 2000–2024.

The dominance of A61 and C07 suggests that a substantial part of the filing corpus was located in medical, hygiene, pharmaceutical, and organic-chemistry-related areas at the IPC class level. However, this interpretation should remain descriptive because the present analysis is based on primary IPC classes and does not decompose the corpus by applicant origin, patent family, subclass, claim content, or assignee sector. Therefore, the large shares of A61 and C07 should be interpreted as evidence of concentration in these IPC-classified filing domains before INDECOPI, not as direct evidence of domestic pharmaceutical or biomedical technological capability.

The remaining leading classes indicate that the high-share segment of the corpus extended beyond medical and organic chemistry fields. The Top 15 IPC classes accounted for 72.6% of the panel, confirming that cumulative dominance persisted despite the period-level decline in concentration reported above. These results show that diversification over time occurred within a filing corpus that remained cumulatively anchored in a limited set of high-share IPC classes.

### 3.4 Persistence and distributional change

Table 5 and Figure 4 summarize rank persistence, Top-k overlap, and distributional change across consecutive 5-year IPC-class distributions. In the main fixed-universe specification, computed over the 131 primary IPC classes observed in the 2000–2024 analytical panel, Spearman's rho remained high across all consecutive period pairs. It was 0.894 between 2000–2004 and 2005–2009, 0.887 between 2005–2009 and 2010–2014, 0.940 between 2010–2014 and 2015–2019, and 0.941 between 2015–2019 and 2020–2024. Kendall's tau-b provided a consistent complementary result, ranging from 0.734 to 0.822. Rank-correlation *p*-values were reported as thresholds, with all consecutive comparisons showing *p* < 0.001.

**Table 5:** Rank persistence, Top-k overlap, and Jensen–Shannon distance across consecutive IPC-class distributions, 2000–2024.

First period	Second period	Spearman's ρ	Spearman *p*-value	Kendall's τ-b	Kendall *p*-value	Jaccard top-5	Jaccard top-10	Jaccard top-20	Jensen–Shannon distance	Shared top-10 IPC classes	Exited top-10	Entered top-10
2000–2004	2005–2009	0.894	<0.001	0.756	<0.001	0.429	0.667	0.818	0.161	A01, A23, A61, B01, B65, C07, C12, C22	A47, G06	G01, H04
2005–2009	2010–2014	0.887	<0.001	0.734	<0.001	0.429	0.667	0.6	0.236	A01, A23, A61, B01, B65, C07, C12, C22	G01, H04	E04, E21
2010–2014	2015–2019	0.94	<0.001	0.822	<0.001	1	0.667	0.739	0.188	A01, A23, A61, B01, B65, C07, C12, C22	E04, E21	A47, G01
2015–2019	2020–2024	0.941	<0.001	0.811	<0.001	0.429	0.818	0.818	0.134	A01, A23, A47, A61, B01, B65, C07, C12, G01	C22	E02

Metrics were computed over the fixed universe of 131 primary IPC classes observed in the 2000–2024 analytical panel. Rank-correlation *p*-values are reported as thresholds and are interpreted as complementary information. Full robustness results across alternative IPC-class universes are reported in Supplementary Table S3.

**Figure 4:**
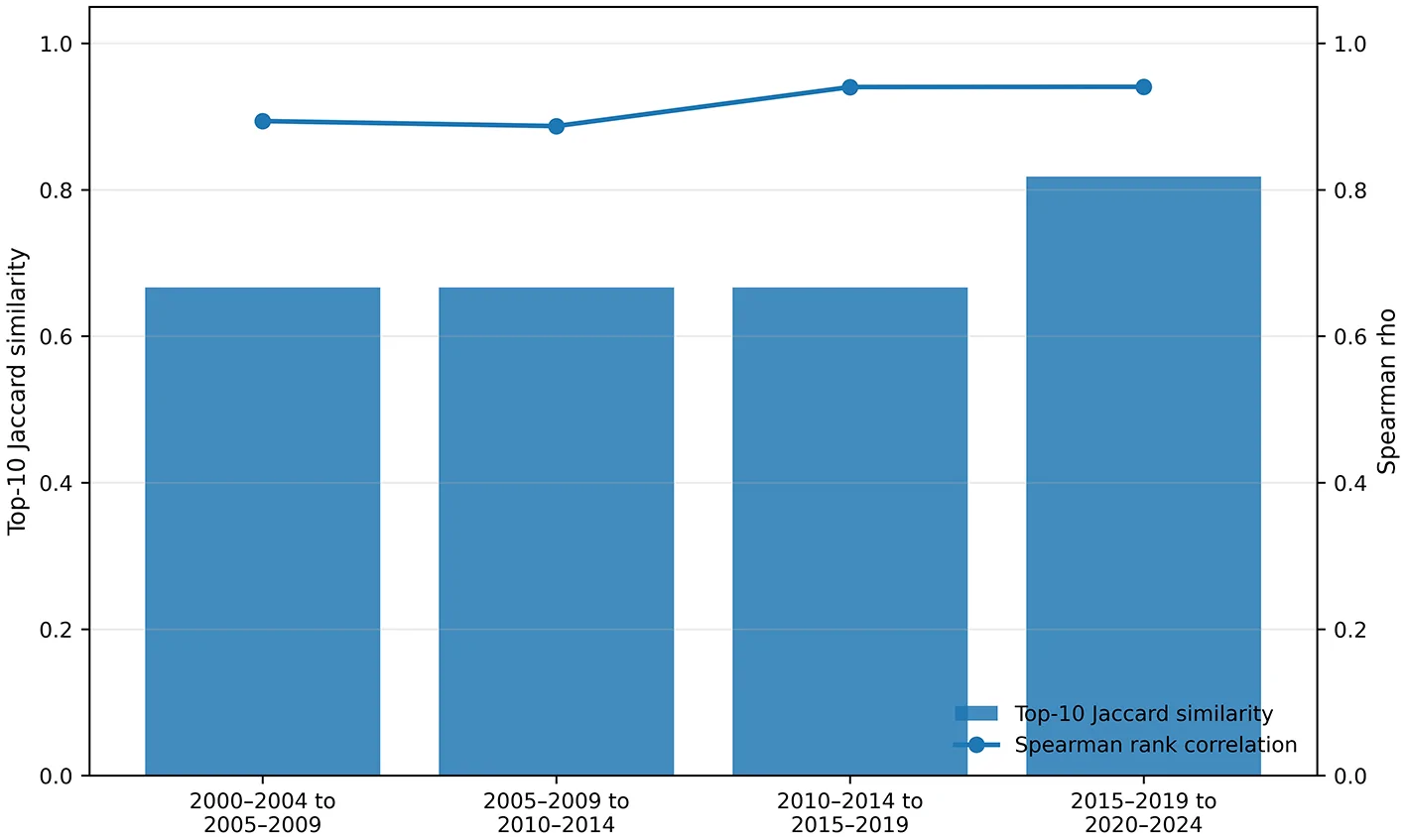
Rank persistence and Top-10 Jaccard similarity across consecutive IPC analytical periods, 2000–2024.

Top-k overlap also indicated continuity in the membership of dominant IPC classes. Top-10 Jaccard similarity ranged from 0.667 to 0.818 across consecutive periods. The highest Top-10 overlap occurred in the final transition, with a Jaccard value of 0.818 and only one Top-10 replacement, with C22 exiting and E02 entering. These results show that the leading IPC-class set changed gradually rather than being fully replaced.

Table 6 and Figure 5 present the full pairwise Jensen-Shannon distance matrix across the five analytical periods. Jensen-Shannon distance was used to assess broader compositional change between period-level IPC-share distributions. Consecutive Jensen-Shannon distances were 0.161, 0.236, 0.188, and 0.134, respectively. The smallest consecutive distance occurred between 2015–2019 and 2020–2024, whereas the largest pairwise distance in the full matrix occurred between 2005–2009 and 2020–2024. These distances are interpreted comparatively rather than through uncalibrated qualitative labels.

**Table 6:** Jensen–Shannon distance matrix across IPC-class distributions by analytical period, 2000–2024.

Period	2000–2004	2005–2009	2010–2014	2015–2019	2020–2024
2000–2004	0	0.161	0.236	0.285	0.303
2005–2009	0.161	0	0.236	0.32	0.323
2010–2014	0.236	0.236	0	0.188	0.186
2015–2019	0.285	0.32	0.188	0	0.134
2020–2024	0.303	0.323	0.186	0.134	0

**Figure 5:**
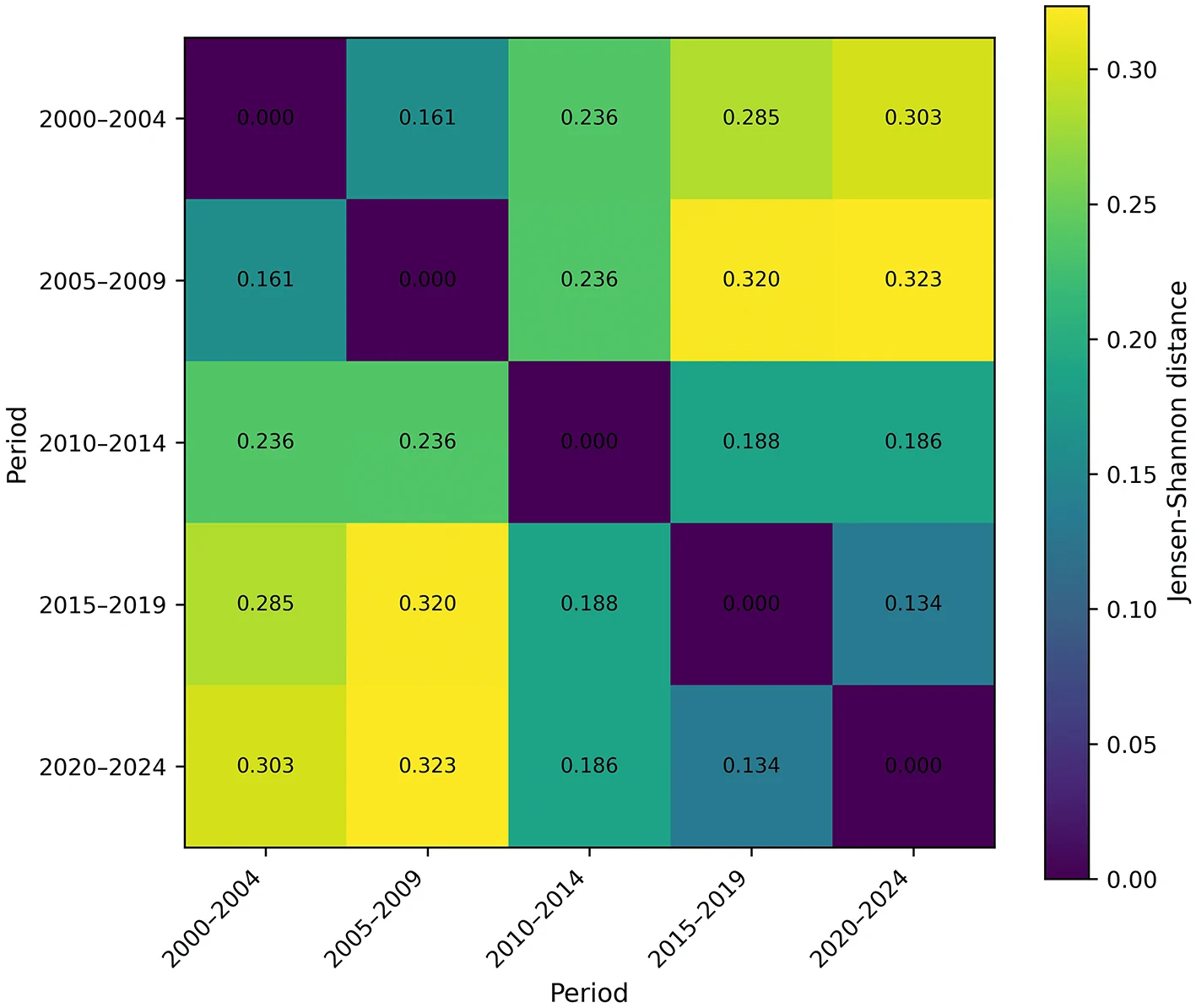
Pairwise Jensen–Shannon distance matrix between IPC-class distributions by analytical period, 2000–2024.

Because fixed-universe rank correlations can overstate persistence when many inactive or low-share classes remain in the vector, robustness checks were conducted using alternative class universes. Supplementary Table S3 summarizes the coefficient ranges across these specifications. Spearman's rho remained positive and substantial under all specifications, but its range decreased when the analysis was restricted to active, intersection-active, Top-k, or filing-threshold class sets. Under the union-active specification, Spearman's rho ranged from 0.868 to 0.930, and under the intersection-active specification it ranged from 0.849 to 0.921. In the Top-20 union specification, Spearman's rho ranged from 0.773 to 0.867, while in the Top-50 union specification it ranged from 0.788 to 0.903. Filing-threshold specifications also supported the same interpretation: Spearman's rho ranged from 0.810 to 0.896 for classes with at least 10 filings across each period pair and from 0.794 to 0.905 for classes with at least 25 filings.

Taken together, the rank-correlation, Top-k overlap, Jensen-Shannon, and robustness results indicate diversification with structural persistence: the IPC-classified filing distribution became broader and less concentrated over time, but the dominant hierarchy changed gradually rather than being fully reordered.

### 3.5 IPC classes with the largest share changes

Table 7 and Figure 6 identify the IPC classes with the largest changes in filing share between the first and last analytical periods. These changes show redistribution within the IPC-classified filing corpus, but they should not be interpreted as direct evidence of technological upgrading, domestic specialization, or sectoral innovation performance. The comparison is based on primary IPC shares in two predefined 5-year periods: 2000–2004 and 2020–2024.

**Table 7:** Largest IPC-class share gains and declines between 2000–2004 and 2020–2024.

IPC class	Domain	Records 2000–2004	Share 2000–2004 (%)	Rank 2000–2004	Records 2020–2024	Share 2020–2024 (%)	Rank 2020–2024	Share change (pp)	Rank change
A01	Agriculture; forestry; animal husbandry	96	1.96	7	540	6.07	3	4.11	4
E02	Hydraulic engineering; foundations	20	0.41	28	207	2.33	9	1.92	19
G01	Measuring; testing	59	1.21	12	264	2.97	6	1.76	6
A47	Furniture; domestic articles	72	1.47	9	269	3.02	5	1.55	4
C12	Biochemistry; microbiology	91	1.86	8	297	3.34	4	1.48	4
E21	Earth or rock drilling; mining	29	0.59	21	167	1.88	12	1.29	9
B02	Crushing; pulverizing; milling	11	0.22	46	119	1.34	16	1.11	30
C22	Metallurgy	129	2.64	4	147	1.65	14	−0.98	−10
C07	Organic chemistry	972	19.86	2	1,369	15.39	2	−4.46	0
A61	Medical or veterinary science; hygiene	1,946	39.75	1	1,612	18.13	1	−21.63	0

Shares are calculated within each analytical period. Share change is reported in percentage points between 2000–2004 and 2020–2024. The table is based on primary IPC classes and does not identify applicant origin, patent value, grant status, or all secondary IPC assignments.

**Figure 6:**
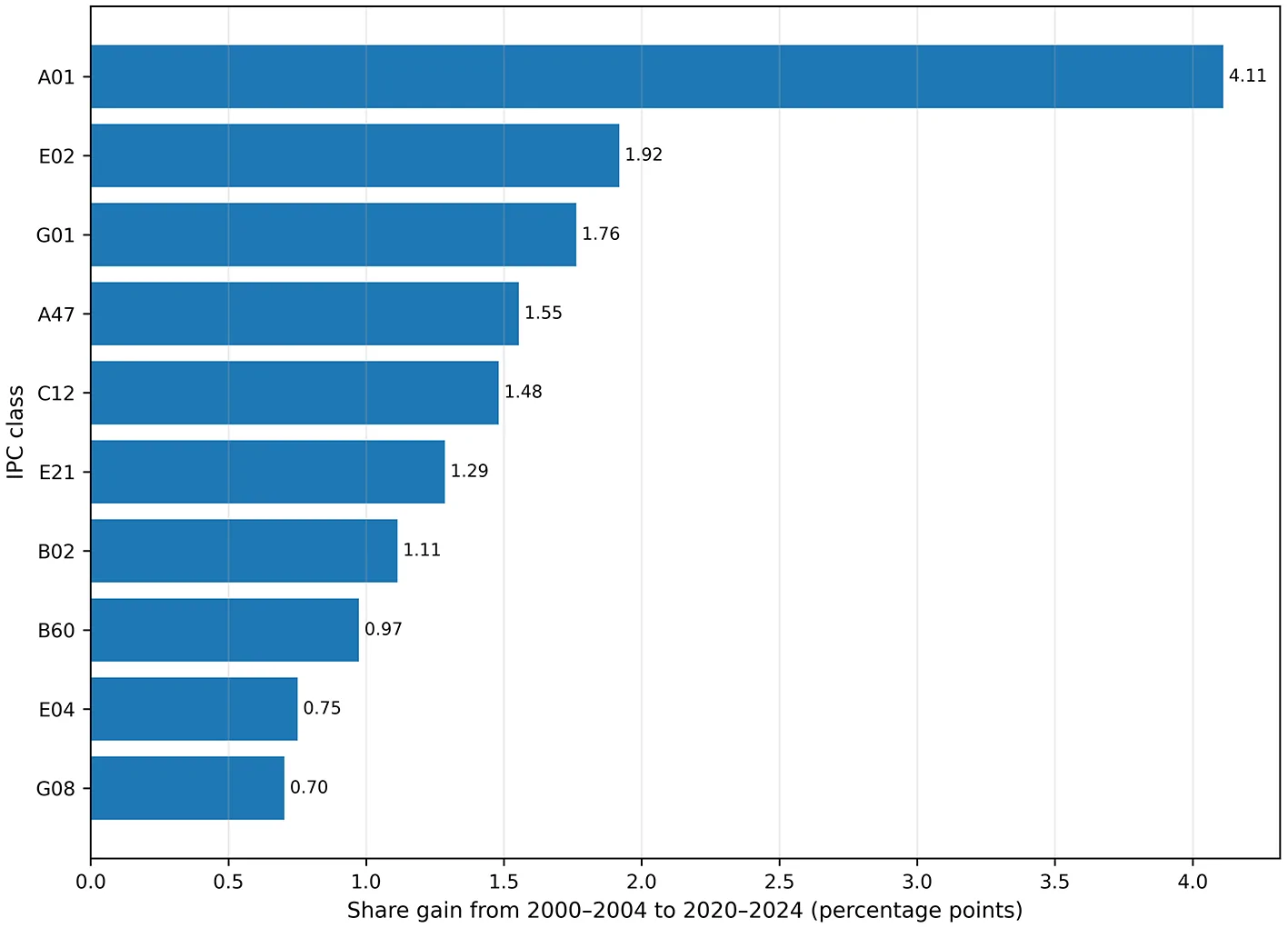
IPC classes with the largest share gains between 2000–2004 and 2020–2024.

A01 showed the largest positive change, increasing from 1.96% of filings in 2000–2004 to 6.07% in 2020–2024, a gain of +4.11 percentage points. Its rank improved from seventh to third. Other classes with gains above one percentage point included E02, G01, A47, C12, E21, and B02.

The largest relative declines occurred in classes that nevertheless remained structurally important. A61 decreased by −21.63 percentage points between 2000–2004 and 2020–2024, but retained rank one in both periods. C07 decreased by −4.46 percentage points and retained rank two. Thus, the main pattern is not one of complete displacement of the leading IPC classes. Instead, A61 and C07 lost relative share while remaining the two highest-ranked classes, and several lower-share classes gained participation in the final period.

### 3.6 Cumulative concentration hierarchy

Table 8 and Figure 7 summarize the cumulative concentration hierarchy of the 2000–2024 analytical panel. The Core zone contains three IPC classes-A61, C07, and A01-which together account for 16,324 records, or 49.5% of the filing corpus, while representing only 2.3% of the 131 IPC classes observed in the panel. Because IPC classes are discrete units, the Core zone does not equal the 50% threshold exactly. It reaches 49.5% because the conservative do-not-cross rule assigns an IPC class to a zone only if its inclusion does not move the cumulative filing share beyond the upper threshold of that zone.

**Table 8:** Cumulative IPC concentration zones in Peru's patent and utility model filing corpus, 2000–2024.

Zone	Number of IPC classes	IPC classes (%)	Records	Corpus share (%)	Illustrative IPC classes
Core (0–50%)	3	2.3	16,324	49.5	A61, C07, A01
Intermediate (50–80%)	19	14.5	9,847	29.9	B65, A23, B01, C12, C22, A47, G01, E21, E04, G06, E02, H04, B60, F16, B02, and others
Peripheral (80–95%)	39	29.8	5,102	15.5	B62, C25, C08, C01, F03, C10, C04, F42, F24, F04, A63, E05, C09, F25, B32, and others
Long-tail (95–100%)	70	53.4	1,693	5.1	D06, B23, G05, H05, F21, A43, B09, B43, F27, F23, B21, D21, A46, D01, F26, and others

Zones were defined by cumulative filing-share thresholds after ranking IPC classes in descending order of total records in the 2000–2024 analytical panel. A conservative do-not-cross rule was applied at each boundary. The labels are descriptive and do not imply a network-based core-periphery model.

**Figure 7:**
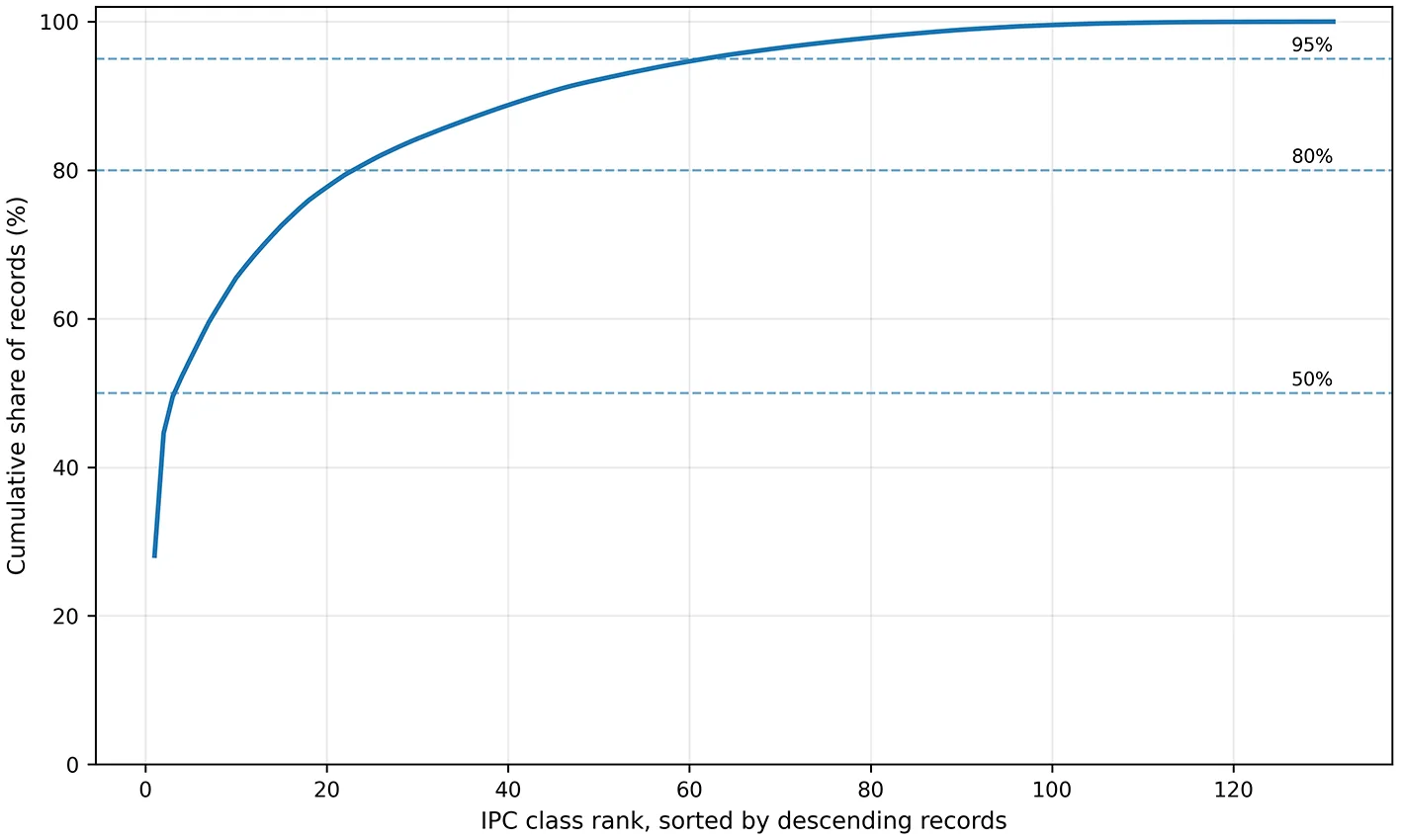
Cumulative IPC concentration zones in Peru's patent and utility model filing corpus, 2000–2024.

The Intermediate zone contains 19 IPC classes and accounts for 9,847 records, or 29.9% of the corpus. Together, the Core and Intermediate zones represent 22 IPC classes, or 16.8% of the observed IPC universe, but account for 79.4% of all filings. The Peripheral and Long-tail zones include 109 classes, representing 83.2% of the IPC universe, but account for only 20.6% of filings.

Thus, although the annual and period-average results showed broader IPC participation and lower concentration over time, the cumulative hierarchy remained highly uneven. The zone labels are descriptive cumulative concentration categories and do not refer to a network-based core-periphery model, statistical clustering, or technology-community structure.

### 3.7 Regional context and applicant-origin interpretation

Table 9 provides regional applicant-origin context using aggregate World Bank World Development Indicators data on patent applications by residents and non-residents. These data are used only to contextualize the INDECOPI filing corpus and do not reproduce the IPC-class analysis. The regional comparison reports annual means for 2000–2005 and the latest available WDI year in the supplied resident and non-resident patent-application series, which was 2021 for the selected countries. Therefore, the most recent comparison is reported as 2021 rather than as a 2021–2024 period.

**Table 9:** Resident and non-resident patent applications in selected Latin American countries: annual means for 2000–2005 and latest available WDI year, 2021.

Country	Window	Resident applications	Non-resident applications	Total patent applications	Resident share (%)	Non-resident/resident ratio
Brazil	2000-2005 annual mean	3,677.20	13,562.70	17,239.80	21.3	3.69
Brazil	2021 latest available WDI year	4,666.00	19,566.00	24,232.00	19.3	4.19
Chile	2000-2005 annual mean	325	2,456.20	2,781.20	11.7	7.56
Chile	2021 latest available WDI year	402	2,680.00	3,082.00	13	6.67
Colombia	2000-2005 annual mean	75.2	1,189.70	1,264.80	5.9	15.83
Colombia	2021 latest available WDI year	432	1,855.00	2,287.00	18.9	4.29
Mexico	2000-2005 annual mean	518	12,736.70	13,254.70	3.9	24.59
Mexico	2021 latest available WDI year	1,117.00	15,044.00	16,161.00	6.9	13.47
Peru	2000-2005 annual mean	33.7	920.3	954	3.5	27.34
Peru	2021 latest available WDI year	94	1,141.00	1,235.00	7.6	12.14

Resident and non-resident patent-application counts were taken from World Bank World Development Indicators series on patent applications by residents and non-residents. The 2000–2005 values are annual means. The latest comparison uses 2021 because the supplied WDI resident and non-resident series did not contain usable observations for 2022–2024 for the selected countries. These aggregate patent-application data do not include utility models and were not merged with the INDECOPI IPC-level analytical panel.

In Peru, resident patent applications increased from an annual mean of 33.7 in 2000–2005 to 94 in 2021, while non-resident applications increased from an annual mean of 920.3 to 1,141 over the same comparison points. The resident share increased from 3.5% to 7.6%, and the non-resident/resident ratio declined from 27.34 to 12.14. This indicates an improvement in resident participation relative to the early 2000s, but also confirms that Peru's patent filing environment remained strongly shaped by non-resident applications in the latest available WDI year.

Compared with the selected Latin American countries, Peru remained at the lower end of resident participation. In 2021, Brazil recorded a resident share of 19.3% and a non-resident/resident ratio of 4.19, while Colombia recorded a resident share of 18.9% and a ratio of 4.29. Chile showed an intermediate pattern, with a resident share of 13.0% and a ratio of 6.67. Mexico was closer to Peru, with a resident share of 6.9% and a non-resident/resident ratio of 13.47.

These regional indicators should be interpreted with caution. They refer to aggregate patent applications as reported in WDI, not to the IPC-classified patent and utility model panel analyzed in the main study. They do not include utility models, do not provide IPC-class distributions, and do not identify whether specific IPC classes in the INDECOPI corpus were driven by resident or non-resident applicants.

## 4 Discussion

### 4.1 Diversification without structural reordering

The main finding of this study is that Peru's patent and utility model filing corpus became less concentrated and more broadly distributed across IPC classes, but this diversification did not amount to full structural reordering. The period-average results provide the strongest evidence for this interpretation. Mean annual HHI declined from 0.220 in 2000–2004 to 0.072 in 2020–2024, while mean Shannon entropy increased from 3.670 to 5.022 bits. Dominance indicators moved in the same direction: mean Top-1 share decreased from 40.54% to 18.21%, mean Top-3 share from 62.74% to 39.79%, and mean Top-10 share from 77.31% to 59.79%. Because these comparisons are based on predefined 5-year windows, they reduce the risk of overinterpreting selected annual peak-to-endpoint changes.

These changes indicate a broader and more even IPC-classified filing distribution. However, they should be interpreted as distributional diversification rather than as evidence of higher technological capability, patent quality, or domestic innovation performance. Patent-based indicators are informative but imperfect measures of technological activity, and their interpretation depends on legal instruments, applicant populations, filing strategies, jurisdictional coverage, and counting conventions. Similarly, HHI, entropy, and Top-k shares summarize the internal structure of a filing distribution; they do not measure the economic value, inventive quality, or resident technological capacity behind the filings (Griliches, 1990; Shannon, 1948; Stirling, 2007).

The distinction between diversification and structural reordering is central to the contribution of this article. Although concentration declined over time, the cumulative hierarchy remained strongly anchored in a small group of IPC classes. A61 and C07 together accounted for 44.6% of the 2000–2024 panel, and A61, C07, and A01 together accounted for 49.5%. This means that broader IPC participation occurred within a corpus that remained cumulatively dominated by medical or veterinary science and hygiene, organic chemistry, and agriculture-related classes. The decline in relative dominance of A61 and C07 therefore did not imply their displacement from the top of the hierarchy.

The persistence results reinforce this interpretation. In the main fixed-universe specification, Spearman rank correlations across consecutive 5-year periods ranged from 0.887 to 0.941, while Top-10 Jaccard similarity ranged from 0.667 to 0.818. However, the revised robustness analysis shows that this finding is not solely an artifact of retaining the full 131-class universe. When the analysis was restricted to alternative class universes, including union-active classes, intersection-active classes, Top-20 and Top-50 classes, and filing-threshold subsets, rank-persistence coefficients decreased as expected but remained consistently positive and substantial. This indicates that persistence was not driven only by inactive or low-share classes in the tail of the distribution.

Jensen-Shannon distances provide a complementary view of compositional change. Consecutive period distances ranged from 0.134 to 0.236, while the largest pairwise distance in the full period matrix occurred between 2005–2009 and 2020–2024. These values are interpreted comparatively rather than through uncalibrated labels. They show that the IPC-share distribution changed over time, but that adjacent-period change was gradual rather than abrupt. Taken together, the evidence supports a pattern of diversification with structural persistence: the filing corpus became broader and less concentrated, but the dominant IPC hierarchy was not fully reordered.

This result is important for patent analytics because it shows why aggregate filing counts or single concentration indicators can be misleading when used alone. A decline in HHI or an increase in entropy may suggest greater breadth, but it does not reveal whether dominant classes have been displaced. Conversely, high rank persistence does not mean that the corpus did not diversify. The integrated framework used here-combining concentration, entropy, Top-k dominance, rank persistence, Top-k overlap, Jensen-Shannon distance, cumulative zones, and instrument-stratified robustness-makes it possible to distinguish broader distributional participation from deeper hierarchical change.

### 4.2 Why applicant origin matters for patent metrics

A second implication of the results is that applicant origin conditions how the IPC structure of a national filing corpus should be interpreted. Applications filed before a national intellectual property office reflect a jurisdictional filing environment, not necessarily the technological profile of resident applicants. This distinction is especially important in patent systems where non-resident applicants account for a large share of patent filings. In such settings, the IPC composition of the office-based corpus may reflect domestic technological activity, foreign protection strategies, or a combination of both. Therefore, the IPC-classified filing distribution analyzed in this study should not be equated with Peru's domestic technological capability.

The regional applicant-origin comparison reinforces this caution. Using World Bank resident and non-resident patent-application series, Peru's resident patent-application share increased from an annual mean of 3.5% in 2000–2005 to 7.6% in 2021, the latest available WDI year in the supplied series. Over the same comparison points, the non-resident/resident ratio decreased from 27.34 to 12.14. This indicates higher resident participation than in the early 2000s, but it also shows that Peru's patent filing environment remained strongly shaped by non-resident applications.

This context is central for interpreting the dominance of A61 and C07 in the INDECOPI filing corpus. At the IPC class level, A61 corresponds to medical or veterinary science and hygiene, while C07 corresponds to organic chemistry. Together, these two classes accounted for 44.6% of the 2000-2024 panel. Their dominance suggests that a substantial part of the filing corpus was located in medical, hygiene, pharmaceutical, and organic-chemistry-related domains. However, because the main analysis does not decompose IPC classes by applicant origin, applicant type, assignee sector, patent family, grant status, claims, or subclass-level detail, this pattern should not be interpreted as direct evidence of domestic pharmaceutical, biomedical, or chemical technological capability.

A01, corresponding to agriculture, forestry, and animal husbandry, requires a similarly cautious interpretation. It was the third-largest IPC class in the cumulative panel and showed the largest positive share change between 2000–2004 and 2020–2024. This is relevant because agriculture-related technological domains are institutionally and economically meaningful in the Peruvian context. Nevertheless, the present evidence only shows that A01 became more prominent in the IPC-classified filing distribution before INDECOPI. It does not establish whether the increase was driven by resident applicants, non-resident applicants, utility model filings, patent filings, specific crop systems, public research organizations, firms, or individual inventors.

The regional comparison is therefore best understood as contextual evidence that strengthens the interpretation of the INDECOPI corpus as a jurisdictional filing corpus. It does not validate or replace the IPC-based analysis, and it does not identify which IPC classes are resident- or non-resident-driven. Future research should link IPC classes with applicant origin, applicant type, legal instrument, grant status, and patent-family information to distinguish domestic inventive activity from foreign filing strategies more directly.

### 4.3 Implications for patent analytics and technology intelligence

The results have methodological implications for patent analytics and technology intelligence. First, they show that aggregate filing counts are insufficient for interpreting the structure of a national patent-office corpus. A filing series can increase in volume while remaining concentrated in a small number of technological classes, and a decline in concentration does not necessarily imply displacement of the dominant hierarchy. For this reason, national filing corpora should be examined through complementary indicators that distinguish filing volume, distributional breadth, dominance, rank persistence, class-set continuity, and compositional distance.

Second, the findings show the value of separating distributional diversification from structural reordering. In the INDECOPI corpus, mean annual HHI declined and Shannon entropy increased across predefined 5-year windows, indicating broader and more even IPC participation. However, A61 and C07 remained the two leading classes, and the Top-10 class membership remained relatively stable across consecutive periods. This combination would be difficult to interpret using only aggregate counts or a single diversity indicator.

Third, the robustness checks illustrate why patent analytics should make counting rules and analytical universes explicit. The primary IPC diagnostic showed that the selected primary class corresponded to the first listed IPC symbol and that the construction was not consistent with a strict alphabetical sorting artifact. The instrument-stratified analysis showed that the main diversification pattern was not only a mechanical result of combining patents and utility models. The persistence robustness analysis showed that rank stability remained evident under alternative class universes, although coefficients decreased when the analysis was restricted to active, Top-k, or filing-threshold subsets.

Fourth, the cumulative-zone analysis provides a simple way to communicate hierarchy without claiming more than the data support. The Core, Intermediate, Peripheral, and Long-tail zones summarize how filings are distributed across high-share and low-share IPC classes. However, the zones should not be interpreted as strategic sectors, emerging technologies, or network communities unless additional evidence is incorporated. For policy or technology-intelligence purposes, such additional evidence could include applicant origin, applicant type, grant status, patent families, claims, citations, maintenance, subclass-level detail, or text-based analysis.

Fifth, the study reinforces the importance of reproducibility in patent analytics. Patent-office administrative datasets often contain restricted microdata, which limits the possibility of sharing raw records publicly. Nevertheless, reproducibility can still be improved by sharing derived aggregate matrices, codebooks, scripts, audit tables, robustness outputs, and clear documentation of counting rules. The framework applied here does not identify patent value, technological quality, or domestic innovation capacity by itself. Its contribution is more specific: it provides a reproducible descriptive structure for distinguishing broader IPC-classified filing participation from deeper changes in technological hierarchy.

### 4.4 Limitations and future research

This study has several limitations that should guide interpretation and future research. First, the main analysis used one primary IPC class per application. This counting rule avoided double counting and allowed mutually exclusive annual and period-level IPC-share distributions, but it did not capture the full set of secondary IPC assignments that may appear in patent and utility model records. As a result, the study describes primary IPC-class filing distributions rather than all-IPC technological profiles, fractional IPC distributions, or co-classification networks.

Second, the administrative extract did not contain explicit WIPO ST.8 invention-information or additional-information flags, nor an independent field identifying a formally designated primary IPC symbol. The primary IPC class was therefore operationalized as the class of the first IPC symbol recorded in the original classification string. Diagnostic checks showed that this rule was reproducible and did not appear to be driven by a strict alphabetical sorting artifact. Nevertheless, the analysis cannot verify whether the first listed IPC symbol corresponds to an administratively designated invention-information symbol in every record.

Third, the study analyzed the jurisdictional filing corpus before INDECOPI and did not decompose IPC classes by applicant origin. The regional comparison showed that Peru's patent filing environment remains strongly shaped by non-resident applications, but the available regional data do not provide IPC-level resident/non-resident distributions. Therefore, the study cannot determine whether dominant IPC classes such as A61, C07, or A01 were mainly driven by resident applicants, non-resident applicants, or both.

Fourth, the analysis focused on filing structure rather than patent outcomes, quality, or economic value. Applications were counted regardless of whether they were eventually granted, abandoned, rejected, maintained, cited, commercialized, or linked to patent families in other jurisdictions. Consequently, the results describe the structure of applications filed before INDECOPI, not the technological value, legal strength, market relevance, or innovation impact of those filings.

Fifth, although the study added instrument-stratified robustness checks for patent applications and utility model applications, the main interpretation still centers on the combined IPC-classified filing corpus. This is appropriate for characterizing the jurisdictional filing structure before INDECOPI, but patents and utility models remain legally and strategically distinct instruments. Future work could examine their differences in greater depth, including applicant composition, IPC specialization, grant or registration outcomes, processing times, and the role of utility models in incremental or locally adapted technological activity.

Sixth, the temporal design used predefined 5-year periods to reduce annual volatility and improve comparability. This choice strengthens the interpretation of long-run distributional change, but it may smooth short-term administrative, regulatory, economic, or sector-specific shocks. Future research could combine the period-level approach with event-sensitive analyses, rolling windows, year-to-year transition matrices, or policy-timeline interpretation, provided that external institutional evidence is available to support causal or contextual claims.

Finally, the analysis depends on the consistency and completeness of the INDECOPI administrative database and the harmonization procedures applied to IPC fields. The study included record-flow audits, IPC-format checks, primary-IPC diagnostics, robustness analyses, and reproducibility materials, but it did not independently reclassify the technical content of each application. Taken together, these limitations define the scope of the findings. The study provides a reproducible descriptive analysis of Peru's IPC-classified patent and utility model filing corpus. It does not measure domestic technological capability, patent quality, technological value, or innovation impact directly.

## 5 Conclusion

This study applied an integrated descriptive patent analytics framework to 32,966 patent and utility model applications filed before Peru's INDECOPI between 2000 and 2024 and classified into 131 primary IPC classes. The results show that the IPC-classified filing distribution became less concentrated, broader, and more even over time. Based on predefined 5-year analytical windows, mean annual HHI declined from 0.220 in 2000–2004 to 0.072 in 2020–2024, while mean Shannon entropy increased from 3.670 to 5.022 bits. Dominance indicators moved in the same direction, with mean Top-1, Top-3, and Top-10 shares all declining between the first and last analytical periods.

The central conclusion is that Peru's patent and utility model filing corpus experienced diversification without full structural reordering. The corpus broadened across IPC classes, and several lower-share classes gained participation, including A01, E02, G01, A47, C12, E21, and B02. However, the cumulative hierarchy remained highly uneven. A61 and C07 together accounted for 44.6% of the 2000–2024 panel, while A61, C07, and A01 together accounted for 49.5%. A61 and C07 lost relative share but retained the first and second ranks, indicating that diversification occurred without complete displacement of the leading IPC classes.

Persistence and robustness analyses support this interpretation. In the main fixed-universe specification, rank correlations across consecutive 5-year periods remained high, while Top-k overlap showed continuity in the membership of dominant IPC classes. Robustness checks using union-active, intersection-active, Top-20, Top-50, and filing-threshold class universes reduced the magnitude of rank-persistence coefficients, as expected, but did not overturn the finding of structural persistence. Jensen-Shannon distances showed gradual compositional change rather than abrupt reorganization.

The findings should be interpreted within the scope of an office-based jurisdictional filing corpus. The analysis does not measure domestic technological capability, patent quality, technological value, or innovation impact directly. This caution is especially important because applications filed before INDECOPI include both resident and non-resident applicants, and the regional applicant-origin comparison indicates that Peru's patent filing environment remained strongly shaped by non-resident applications in the latest available WDI year.

The main contribution of the study is methodological and empirical. Empirically, it provides a longitudinal IPC-based characterization of Peru's patent and utility model filing corpus for 2000–2024. Methodologically, it shows the value of combining concentration, entropy, Top-k dominance, rank persistence, set overlap, Jensen-Shannon distance, cumulative concentration zones, instrument-stratified robustness, primary-IPC diagnostics, and applicant-origin context. This integrated approach helps distinguish broader IPC-classified filing participation from deeper changes in technological hierarchy and can be transferred to other national or regional patent-office datasets with filing-year and IPC information.

## Data Availability

Derived aggregate datasets, code, documentation, and reproducibility materials supporting the findings of this study are available in Zenodo at https://doi.org/10.5281/zenodo.22070845 (all versions), with corrected version v1.1 available at https://doi.org/10.5281/zenodo.22178836. The underlying INDECOPI administrative microdata are subject to institutional access conditions and cannot be publicly redistributed by the authors. Requests for access to the source administrative database should be directed to INDECOPI through the applicable institutional procedures.
